# Supplementary material for: New estimates of genome size in Orthoptera and their evolutionary implications
Source: PLoS One. 2023 Mar 15;18(3):e0275551. doi: 10.1371/journal.pone.0275551 (PMC10016648; doi:10.1371/journal.pone.0275551)
Supplement: Attachment — Submitted filename: Rebuttal.docx [file pone.0275551.s003.docx]

**We thank the editor and both reviewers for the very helpful comments!**

When submitting your revision, we need you to address these additional requirements.

1. Please ensure that your manuscript meets PLOS ONE's style requirements, including those for file naming. The PLOS ONE style templates can be found at

[https://journals.plos.org/plosone/s/file?id=wjVg/PLOSOne_formatting_sample_main_body.pdf](https://deref-gmx.net/mail/client/X9RQVttdVls/dereferrer/?redirectUrl=https%3A%2F%2Fjournals.plos.org%2Fplosone%2Fs%2Ffile%3Fid%3DwjVg%2FPLOSOne_formatting_sample_main_body.pdf)

and [https://journals.plos.org/plosone/s/file?id=ba62/PLOSOne_formatting_sample_title_authors_affiliations.pdf](https://deref-gmx.net/mail/client/Psng0f32Rw8/dereferrer/?redirectUrl=https%3A%2F%2Fjournals.plos.org%2Fplosone%2Fs%2Ffile%3Fid%3Dba62%2FPLOSOne_formatting_sample_title_authors_affiliations.pdf).

Done.

2. Please include your full ethics statement in the ‘Methods’ section of your manuscript file. In your statement, please include the full name of the IRB or ethics committee who approved or waived your study, as well as whether or not you obtained informed written or verbal consent. If consent was waived for your study, please include this information in your statement as well.

We added the following statement: “The Government of Upper Bavaria authorized handling, capture, and killing of the insect specimens used in this study with a permit issued on 15 July 2019. Insects were anesthetized and euthanized using CO2.”

Additional Editor Comments:

In addition, I would like to make some suggestions:

Line 103: the word “sacrifice” refers to religious actions, please change it.

We changed the word to “killing”.

Line 110: Why did the authors use the Pisum sativum as an internal standard rather than an insect? Like Drosophila melanogaster or Tenebrio?

We added the explanation to l. 119: “A plant standard was used to ensure comparability with previously conducted measurements of the same facility (8).”

Line 125: What formula was used to calculate genome sizes?

We added the explanation to l. 119: “Sample genome size was calculated as ratio of known standard genome size to measured peak.”


[Note: HTML markup is below. Please do not edit.]

Reviewers' comments:

Reviewer's Responses to Questions

**Comments to the Author**

5. Review Comments to the Author

Reviewer #1: This study is about variation of genome size across the order Orthoptera, an insect order where genomes are known to vary considerably in size. The study uses existing resources and data and presents new genome size estimates for 50 species of Ensifera and Caelifera, which represents a great addition to the existing data. The authors found a new largest measured genome for all insects in the species Bryodemella tuberculata (Caelifera) and they used these estimates to reconstruct ancestral genome sizes based on a phylogenetic tree reconstructed using mitochondrial genomic data.
The study is well presented and written, and both the new data and the conclusions are worth publication. The discussion in itself, reviewing the different causes tested or invoked to explain differences in genome sizes, is very interesting.
I have a few issues about the phylogenetic aspects of the study though, in terms of methodology:

1) The phylogenetic analysis is quite basic, but ML with IQ tree is usually reliable, which makes it acceptable as a reference, but this is not surprising that the tree is not completely resolved. It would have been better to reconstruct the phylogeny using missing data for some species, but keeping the whole mitogenomes when avaiblable in order to maximize the informative content of the data set. This may not change drasticaly the results, which are coherent with previous phylogenies, but it could solve an few uncertainties mentionned by the authors.

We preferred to use the reduced mitochondrial dataset to achieve most possible congruence among the data of the different species. This follows papers of, e.g., Song et al (see references).

2) One important detail is missing in the materials and method section: did the authors partitioned their data set by gene (recommended), or did they consider only one partition for the whole set of genes?

We added to l. 165: “treating all mitochondrial genes as one partition”. As all genes are mitochondrial and linked, we consider this approach as suitable.

3) There is potentially a problem in the study about the comparative methods used to infer ancestral states: the tree from the phylogenetic analysis usually have to be ultrametric to be used for ancestral state reconstructions, which is not the case with trees coming directly from IQtree. A dating analysis or a step of ultrametrisation has be be added in order to respect this principle in phylogenetic comparative analyses.

We added to l. 168: “The ape package implements non-parametric rate smoothing and does not require ultrametrization of the tree.”

4) Another problem in the manuscript is the absence of detailed methods explaining the methods used to infer ancestral states, and why some species present two values in the phylogeny for a given species. Citing the packages is not enough, there should be details about how analyseses were made (which functions and options) and a could be script given in supplemntary material ideally.

Following this comment and the comment by reviewer #2, we uploaded the R code to https://github.com/laradey/Genome_size_Orthoptera.git. This will make it easier for readers to follow the exact workflow we used.

Reviewer #2: Review of Manuscript: PONE-D-22-25859, Hawlitschek et al 2023


General comments on the manuscript:
The authors present a well designed study with clearly stated goals and these goals are thoroughly explored throughout the manuscript. Additionally, I strongly recommend to also submit the genome size estimates to Animal Genome Size Database.

Lines 158-161:
These lines suggest that the authors coded an analysis in the R environment using ‘ape’ and ‘phytools’ packages. It would be ideal if the code and data could be deposited in appropriate data repositories (for example see: data dryrad, [https://datadryad.org/](https://deref-gmx.net/mail/client/JNS57DOx0Zw/dereferrer/?redirectUrl=https%3A%2F%2Fdatadryad.org%2F)). Furthermore, other data and code generated from and during this study can also be archived.

We uploaded the data to github and added the link to the manuscript.

Minor edits:
Line 139: “As this test resulted non-significant (Results),” should perhaps be: “As this resulted in non-significant results,”

Changed to: “As this test resulted yielded non-significant results (see Results)”

Recommendation:
I recommend to publish this study given the minor edits and suggestions are implemented.
